# SYSTEMIZATION OF LAPAROSCOPIC INGUINAL HERNIA REPAIR (TAPP) BASED ON A NEW ANATOMICAL CONCEPT: INVERTED Y AND FIVE TRIANGLES

**DOI:** 10.1590/0102-672020180001e1426

**Published:** 2019-02-07

**Authors:** Marcelo FURTADO, Christiano M. P. CLAUS, Leandro Totti CAVAZZOLA, Flavio MALCHER, Alexandre BAKONYI-NETO, Rogério SAAD-HOSSNE

**Affiliations:** 1 Videolaparoscopic Surgery Service, Pintagueiras Hospital, Jundiaí, São Paulo, Brazil;; 2 Department of Surgical Clinic and Post-Graduation in Mini Invasive Surgery, Positivo University - Jacques Perissat Institute, Curitiba, PR, Brazil;; 3 Department of General Surgery, Hospital de Clínicas, Porto Alegre, RS, Brazil;; 4 Department of Surgery, Celebration Health Florida Center, Florida, USA;; 5 Department of Surgery, Hospital das Clínicas, Botucatu Medical School, Botucatu, SP, Brazil)

**Keywords:** Inguinal herni, Laparoscop, TAP, General surgery, Hérnia Inguina, Laparoscopi, Cirurgia geral.

## Abstract

**Background:**

Laparoscopic inguinal hernia repair has been shown to be superior than open repairs with faster return to daily activities and decrease in the occurrence of chronic pain. However, higher direct costs and mandatory use of general anesthesia are arguments against their use. In addition, increased complexity of surgery resulting from an anatomy that is unusual to general surgeons prevents the widespread adoption of laparoscopic approach.

**Aim:**

To propose a technical systematization for transabdominal laparoscopic repair (TAPP) of inguinal hernias based on anatomical concepts.

**Method:**

To offer a systematization of TAPP repair based on well defined anatomic landmarks, describing the concept of “inverted Y”, identification of five triangles and three zones of dissection, to achieve the “critical view of safety” for laparoscopic inguinal hernia repair.

**Results:**

Since this standardization was developed five years ago, many surgeons were trained following these precepts. Reproducibility is high, as far as, it´s rate of adoption among surgeons.

**Conclusion:**

The concept of the “inverted Y”, “Five triangles” and the dissection based in “Three Zones” establish an effective and reproducible standardization of the TAPP technique.

## RESUMO

**Racional:**

O reparo laparoscópico da hérnia inguinal tem se mostrado superior aos reparos abertos, com retorno mais rápido às atividades diárias e diminuição na ocorrência de dor crônica. No entanto, custos diretos mais altos e o uso obrigatório de anestesia geral são argumentos contra seu uso. Além disso, o aumento da complexidade da operação resultante de uma anatomia incomum aos cirurgiões gerais impede a ampla adoção da abordagem laparoscópica.

**Objetivo:**

Propor uma sistematização técnica para reparo laparoscópico transabdominal (TAPP) de hérnias inguinais com base em conceitos anatômicos.

**Método:**

Oferecer sistematização do reparo do TAPP baseado em pontos anatômicos bem definidos, descrevendo o conceito de “Y invertido”, identificação de cinco triângulos e três zonas de dissecação, para alcançar a “visão crítica de segurança” para o reparo de hérnia inguinal laparoscópica.

**Resultados:**

Desde que essa padronização foi desenvolvida há cinco anos, muitos cirurgiões foram treinados seguindo esses preceitos. A reprodutibilidade é muito alta, assim como a taxa de adoção entre cirurgiões.

**Conclusão:**

O conceito de “Y invertido”, dos “Cinco triângulos” e a dissecção baseada em “Três Zonas” estabelecem uma padronização efetiva e reprodutível da técnica TAPP.

## INTRODUCTION

Repair of inguinal hernias is one of the most common procedures performed by general surgeons around the world^4^ ^,^ ^24^. Although first described in the 1990s, laparoscopic inguinal repair still finds resistance among surgeons today^2^ ^,^ ^7^ ^,^ ^23^. Main reasons are higher direct cost, need for general anesthesia and eventual higher rate of major complications associated with laparoscopic repairs^17^ ^,^ ^22^. Another difficulty related to laparoscopic approach is the greater surgical complexity associated with the need to identify a “new” anatomy of posterior inguinal wall, which is not usual for general surgeons^8^ ^,^ ^17^. Specific training is required to acquire proficiency. 

However, there is current evidence for laparoscopic repairs demonstrating significant advantages such as less complications, especially on recurrent cases, faster recovery and less postoperative chronic pain, in addition to recurrence rates at least equivalent to conventional repairs^6^ ^,^ ^13^ ^,^ ^16^ ^,^ ^18^. Patients have better quality of life scores and degree of satisfaction after laparoscopic inguinal repair, what makes this operation an appropriate treatment for patients with inguinal hernia^1^ ^,^ ^16^.

The aim of this study was to propose a systematization of the transabdominal approach (TAPP) for inguinal repairs with emphasis on dissection in three zones, based on posterior anatomy of the inguinal region and in a didactic definition of what is called “Inverted Y” and “Five Triangles”. 

## METHOD

### Anatomical landmarks

#### Fruchaud miopectineal orifice

**FIGURE 1:**
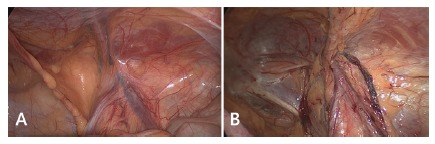
Laparoscopic view of male posterior wall, right inguinal region: A) intact peritoneum; B) dissected peritoneum.

It was described by Fruchaud in 1956 corresponds to the common locations for rising of all hernias in the inguino-crural region, being delimited medially by rectus abdominis muscle, inferiorly by pectineum ligament, laterally by psoas muscle and superiorly by transversus abdominis and internal oblique muscles (transverse arch)^11^. Laparoscopic view, in transabdominal approach, of the posterior inguinal region allows for an easy understanding of this anatomy (Figure 1).

#### A new concept: the “Inverted Y”

**FIGURE 2:**
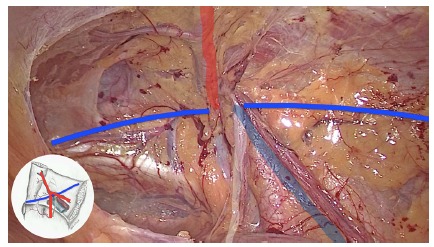
“Inverted Y”: in red inferior epigastric vessels; in white vas deferens; in blue spermatic vessels

To facilitate comprehension and recognition of anatomical structures, the image of an inverted Y in the inguinal region is created with the following elements: inferior epigastric vessels (superiorly), vas deferens (medially) and spermatic vessels (laterally, Figure 2). Recognition of these elements is the basis for understanding the technical steps for repairs all types of inguinal hernias by laparoscopy.

**FIGURE 3:**
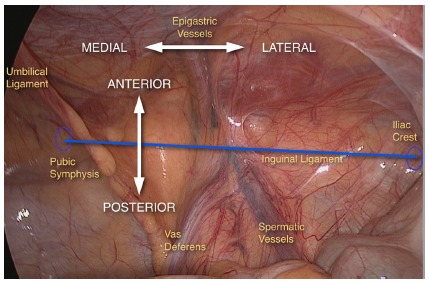
Division of inguinal region in medial and lateral, and anterior and posterior, from inferior epigastric vessels and iliopubic tract (in blue), respectively

Inferior epigastric vessels divide the medial and lateral inguinal regions, defining classification of inguinal hernias as direct (weakness of the transversalis fascia in Hesselbach triangle, medially), or indirect (enlargement of deep inguinal ring, laterally, Figure 3).

Another important anatomical element is the iliopubic tract, which represents the internal view of inguinal ligament. It extends from the anterosuperior iliac crest to the pectineum (Cooper’s) ligament and divides the anterior and posterior inguinal space (Figure 3). Anterior portion is the site of occurrence of inguinal hernias (direct, indirect, and mixed). Femoral or crural hernias, as well as the obturators, are located in the inferior portion of the inguinal space, below the inguinal ligament (and consequently of iliopubic tract)

#### Facilitating anatomical recognition: “the Five Triangles”

**FIGURE 4:**
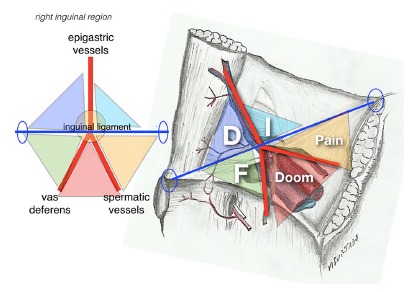
Illustration of “five triangles”: A) illustration of the iliopubic tract crossing the inverted Y and formation of the five triangles; B) anatomical scheme of the inverted Y formed by inferior epigastric vessels, vas deferens and spermatic vessels. The figure also illustrates in a didactic way the representation of five triangles, clockwise: indirect hernias (I), pain (P), doom (D), femoral (F) and direct (D) hernias.

Identification of inverted Y elements and iliopubic tract, that passes horizontally through the deep inguinal ring at the center of the inverted Y, permit visualization of five areas that are, didactically called the “Five Triangles” according to Figure 4.

### Anatomical definitions

#### “Disaster” or “Doom” triangle (or iliac vessels)

Formed by vas deferens medially, and spermatic vessels, laterally, it corresponds to the location of the external iliac vessels (external iliac artery and vein).

#### “Pain” triangle (or of the nerves)

Medially delimited by spermatic vessels and iliopubic tract laterally and superiorly, it represents the passage of lateral cutaneous nerve of the thigh, femoral branch of the genitofemoral nerve and femoral nerve. Recent anatomical studies suggest that the laterosuperior limit should be modified. In a study with cadaveric dissection carried out by Wolfgang et al^19^, it was shown that nerve branches could cross up to 2 cm above the iliopubic tract. It is suggested that this is the new laterosuperior border of pain triangle (2 cm above the iliopubic tract).

#### Triangle of indirect hernias

It is not a true triangle, but it corresponds to the deep inguinal ring, the source of indirect hernias. It is formed by inferior epigastric vessels medially and by iliopubic tract inferiorlaterally.

#### Hesselbach’s triangle or direct hernias

Limits are: medial-lateral border of the rectus abdominis; lateral-inferior epigastric vessels and inguinal ligament (iliopubic tract) inferiorly. It is the site of occurrence of direct hernias. 

#### Triangle of femoral hernias

Again, this is not a true triangle, but identifies the area corresponding to the femoral hernias near the femoral vein ostium, delimited by iliopubic tract superiorly, external iliac vein laterally, pectineum ligament inferiorly and lacunar ligament medially.

**FIGURE 5:**
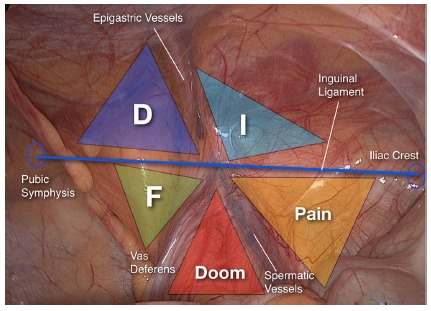
Illustration of anatomical proposal of “Inverted Y and the Five Triangles” over the laparoscopic view, posterior right inguinal floor, in male, with peritoneum still intact

This didactic way of posterior visualization of the myopectineal orifice, defining the inverted Y and the five triangles, facilitates the anatomical understanding of inguinocrural region and of all hernia defects that may occur. In addition, from identification of all key structures it’s possible to establish a technical systematization for dissection of inguinal floor and consequently hernias repairs (Figure 5). Felix and Daes ^9^ described the Critical View of Safety in laparoscopic inguinal hernia repair recently^ ^ in analogy with the to the concept used to reliably perform a laparoscopic cholecystectomy. The “inverted Y and the five triangles” can be used to facilitate the identification of the anatomical landmarks to achieve the described “critical view of safety” for laparoscopic repair of inguinal hernias.

### Technical systematization proposal: The three dissection zones

Based on the concepts above, we created a systematization of the pre-peritoneal space dissection, common to all inguinal repairs performed by TAPP approach. 

### Surgical technique

#### Creation of peritoneal flap

Peritoneal incision is made from medial umbilical ligament, elliptically following the arch of transverse muscle, extending to the anterior superior iliac spine. It can be done from medial to lateral or from lateral to medial. It is important to start at least 4 cm above the deep inguinal ring border to allow the placement of a large prosthesis in the pre-peritoneal space, with overlap of at least 3-4 cm in relation to all potential hernia sites. In addition, this recommendation in the opening flap helps at the end when the peritoneal flap should be closed, covering completely the mesh without contact with intraperitoneal organs.

**FIGURE 6:**
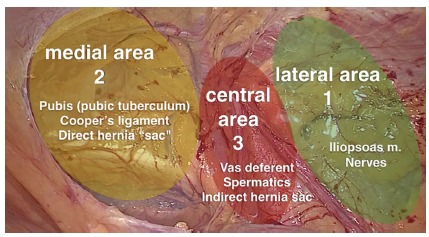
Zones of dissection of pre-peritoneal space following the tactical proposal for standardization of TAPP technique

From this point, we define three areas of dissection called Zones 1, 2 and 3 (Figure 6).

#### Zone 1

Corresponds to the lateral area to inferior epigastric vessels and spermatic vessels. Opening of peritoneum in this area is performed by traction of peritoneum and counter-traction or “parietalization” of the pre-peritoneal fat that covers the lateral cutaneous nerve of the thigh, femoral nerve and branches of the femoral genital nerve. The plane that exposes the muscle in lateral dissection should be avoided. Fatty tissue present in the pre-peritoneal space should be kept in contact with the inguinal floor and not with the peritoneum, thus reducing the risk of manipulation and eventual nerve damage. The lateral border of this dissection is the anterosuperior iliac spine and psoas muscle represents the posterior limit of the dissection. In addition to psoas muscle, the spermatic vessels are identified. Nerves should not be sought in systematic way in order to avoid injury.

#### Zone 2

Is medial to inferior epigastric vessels and corresponds to the site of direct hernias. Dissection of zone 2 should extend to the entire pre-vesical (or Retzius) space until identification of pectineum ligament (Cooper’s) and pubic symphysis. Generally is performed by blunt dissection because there is loose areolar tissue. One should extend the dissection up to midline (pubic symphysis) and 1-2 cm beyond, and 1-2 cm below the pubis in order to create sufficient space for accommodation of adequate sized mesh. A direct hernia defect, when present, is found medially to the epigastric vessels and above the pectineum ligament. In this dissection, the hernia “pseudo-sac” is characterized by weakness of the transversalis fascia and its content, which is usually composed of pre-peritoneal fat. Hernia content is then mobilized, by traction and contraction, of the transversal fascia (which returns to the inguinal canal floor) by exposing the orifice or hernia orifice. The traction and fixation of the weakened transverse fascia in the pectine ligament or even in the rectus abdominis have been advocated by some authors in order to decrease seroma formation in the “dead space” produced by the reduction of the hernia content.

#### Zone 3

Corresponds to the operative step that demands more attention because it is the mobilization of the peritoneum over the vas deferens and spermatic vessels, a region where most commonly are located the external iliac vessels. Dissection of indirect hernia sac at this point is the most demanding step in laparoscopic correction of inguinal hernia and is best performed after the medial and lateral dissection (Zones 1 and 2). This triangle formed medially by vas deferens, laterally by spermatic vessels and inferiorly by psoas muscle, determines the area of ​​insertion of the iliac vessels, artery and vein, named danger triangle or doom (death). In this moment, the peritoneum of these structures is removed by means of traction of the peritoneal flap and counter-traction of the elements of the spermatic cord to the abdominal wall, a movement that can be called “parietalization of the elements of the spermatic funiculus”. Peritoneum flap can be mobilized laterally or medially, in order to allow visualization of the elements that lie posteriorly. In women, the round ligament of the uterus is usually closely adhered to the peritoneum, making its detachment in many cases time consuming. Transection of round ligament is then recommended adjacent to its insertion into the deep inguinal ring, thereby facilitating the continuation of the peritoneum detachment more deeply. All attention must be made to avoid injury of the genital branch of the genitofemoral nerve at this location. In indirect hernias, the hernia sac located anteriorly and laterally to the spermatic vessels and can be easily dissected into the peritoneal cavity and isolated from the cord elements. In the inguino-scrotal hernias the peritoneum enters the deep inguinal ring and can communicate with the vaginal tunic in the scrotum, through the inguinal canal to the testis. Release of this peritoneal leaflet next to the spermatic vessels and vas deferens and consequent reduction of the hernia sac is often hampered by fibrosis of the peritoneal tissue and dense adhesions to the vessels. In these cases, a circular incision of the peritoneum may be performed near the deep inguinal ring. In this way, the hernia sac, which enters the inguinal canal, is abandoned, what facilitates the procedure and, consequently, reduces the risk of inadvertent injury of the elements of the spermatic funiculus, reducing the risk of ischemic orchitis, inguinal scrotal hematoma and/or testicular atrophy. However, the incidence of inguino-scrotal seroma or “pseudo-hydrocele” is greater when this maneuver is adopted. In these particular situations, patients should be warned about fact and advised that in general seroma is reabsorbed after 8-12 weeks.

The dissection of the peritoneum and pre-peritoneal space is given as complete when the elements that make up the inverted Y are visualized as well as the iliopsoas (posterior), pubis and Cooper (medial).

Once the pre-peritoneal space has been adequately dissected, it is easy to place a large prosthesis (usually at least 11-12 cm craniocaudally x 15 cm laterolaterally), covering all areas of weakness of inguinal region with overlap of at least 3-4 cm. The mesh should reach medially at least the pubic symphysis and laterally the iliopsoas muscle. Inferiorly it should descend 1-2 cm below the pubis and superiorly cover 3-4 cm the anterior abdominal wall in relation to the deep inguinal annulus.

The standardization of mesh fixation, if used, must obey the rules below: 1) avoid bone structures: tacking should be performed above the pubic bone, thus avoiding the risk of chronic osteitis; 2) attention to the path of inferior epigastric vessels; 3) staples should not be placed in the triangles of disaster and pain (consider 2 cm above the iliopubic tract as a security area for stapling, in view of current literature evidence regarding the position of the nerves); 4) 5-6 shots are sufficient to fix the mesh (higher shot number is associated with increased risk of chronic pain)^3^.

Peritoneal closure should cover the mesh in order to avoid contact with the intraperitoneal structures, as well as be performed in a way to avoid gaps, either between the staples or sutures, that may be the site of the bowel herniation, that could lead to an intestinal obstruction. Another concern of peritoneum closure is that it should not fold the inferior portion of the mesh, potential cause of recurrence. Wide inferior dissection of the peritoneum avoids this complication. Although technically more difficult, suturing of the peritoneal flap with the use of absorbable sutures is our preferred method of peritoneal closure. Barbed sutures, when correctly used, can facilitate this task.

## RESULTS

This standardization technique was initially used by a single surgeon, who between 1996 and 2010 operated 616 patients (829 hernias). One hundred and forty-one (22.9%) operations were carried out in recurrence hernias. Operative time during learning curve (first 50 cases) ranged from 80 to 130 min for the unilateral and bilateral, respectively. After the first 100 cases operative time was 41 min for unilateral hernias and 63 min to the bilateral ones. Conversion to open repair was needed in two cases (0.32%). Vast majority of patients (99.7%) were discharged within 24 h

### Intraoperative complications

Overall rate of perioperative complications was 0.8% Epigastric artery injury occurred in three patients (0.4%); two (0.3%) suffered damage to the vas deferens; one patient presented significant bleeding in the inguinal ligament (Cooper) when the mesh was fixed by metallic clips. The last major intraoperative complication was a bladder injury corrected through simple closure.

### Postoperative complications

Overall rate of postoperative complications was 5.5%. Recurrence rate was 0,65% (n=4) and chronic pain happened in three patients (0.4%), in a median follow-up of 12 months.

Based on the good results with this standardization of the technique, a group of surgeons started to use it on a regular basis. As well, in several courses for the teaching of surgeons this systematization was adopted. The technique has proved to be effective and reproducible. 

## DISCUSSION

Laparoscopic techniques for correction of the inguinal hernia are seen as complex surgeries, even by more experienced surgeons. They require a longer learning curve due to the need for knowledge of an anatomy not usual for general surgeons (anatomy of posterior inguinal region) and the lack of technical systematization, which can lead to complications. However, in recent years, many studies including randomized trials have been published reporting significant advantages of laparoscopic approach over conventional repairs such as less postoperative pain and complications, faster recovery, reduced chronic pain and recurrence rate. These data are encouraging more and more surgeons to seek training and adopt laparoscopic repairs.

In order to facilitate teaching and improve safety and results of laparoscopic repairs, recently Felix and Daes^9^ described 10 steps to achieve the Critical View of Safety in laparoscopic inguinal hernia repair, in analogy with the concept used to reliably perform a laparoscopic cholecystectomy. Similarly, systematization of TAPP technique proposal in our study has the objective of standardizing surgery. 

Technical criteria regarding choice of the mesh, use or not of fixation and peritoneal closure were not addressed in the present study, considering objective is to emphasize the importance of anatomical recognition and dissection of the landmarks to avoid complications^9^ ^,^ ^15^ ^,^ ^16^. We just detailed the care that must be taken to fix the mesh with tacks, most commonly used method, and correlate with inverted Y and the five triangles. Atraumatic fixation of the mesh (glues or fibrin sealants and self-gripping meshes) has been advocated by many authors and be a good option to avoid complications specific related to tacks, especially chronic pain^18^ ^,^ ^19^ ^,^ ^20^. As well, it does not appear to be associated with an increased risk of recurrence and may be associated with a lower risk of chronic pain^20,21.^ Initially the most common method of closure was through the use of staples or tackers. In relation to this type of closure, it is important to draw attention to the anatomical references, especially inferior epigastric vessels medially. In addition, lateral to epigastric vessels even superiorly to the triangle of pain, tacking in this region can cause damage to the iliohypogastric and ilioinguinal nerves, which have a path in the anterior wall of the abdomen. In general, 4-5 tackers are sufficient to accomplish this closure. Due to the potential risk of increased postoperative pain associated with the application of tackers to peritoneal closure, many authors recommend closure with suture, usually continuous and with absorbable sutures. Although technically more difficult, suturing in the “roof” of the operative field and under some tension, this form has gained more acceptances among surgeons^22^. Barbed sutures, when correctly used, can facilitate this closure.

Systematization TAPP technique proposal in this study is based on anatomical concepts (view of posterior inguinal floor) associated with the technical knowledge acquired in the last 20 years of experience with routine indication of laparoscopy. The aims were to establish an operative strategy that facilitates the understanding and interpretation of anatomical variables and physiopathology of the hernia itself and to achieve the “critical view of safety” for laparoscopic inguinal repair. As well as, to promote diffusion of the technique in the surgical community.

## CONCLUSION

The concept of the “Inverted Y” and the didactic anatomical “Five Triangles” associated with the proposal of dissection based on “Three Zones”, meets the need to establish a standardization of the TAPP technique, seeking excellence in results of the treatment of inguinal hernia. Furthermore, the step-by-step proposal is easily reproducible.
